# Supplementary material for: Longitudinal assessment of COVID-19 vaccine uptake: A two-wave survey of a nationally representative U.S. sample
Source: PLoS One. 2023 Oct 5;18(10):e0289541. doi: 10.1371/journal.pone.0289541 (PMC10553259; doi:10.1371/journal.pone.0289541)
Supplement: S1 File — (DOCX) [file pone.0289541.s001.docx]

**We are going to ask you some questions about vaccines in general.**

These next questions ask about your opinions regarding vaccines in general. Please read each statement and indicate how much you agree or disagree with it by selecting the appropriate response.

1. I like the idea of vaccines.

Strongly disagree

Disagree

Neither disagree nor agree

Agree

Strongly agree

1. Vaccines are generally safe.

Strongly disagree

Disagree

Neither disagree nor agree

Agree

Strongly agree

1. Vaccines are a way to take good care of myself now and in the future.

Strongly disagree

Disagree

Neither disagree nor agree

Agree

Strongly agree

1. Vaccines are effective

Strongly disagree

Disagree

Neither disagree nor agree

Agree

Strongly agree

1. I get vaccinated because I can also protect people with a weaker immune system.

Strongly disagree

Disagree

Neither disagree nor agree

Agree

Strongly agree

1. Vaccination is a collective action to prevent the spread of diseases.

Strongly disagree

Disagree

Neither disagree nor agree

Agree

Strongly agree

**We are going to ask about your feelings today regarding COVID- 19. Indicate how much you agree or disagree with the following statements.**

- 1. I am scared about getting infected with COVID-19.

Strongly disagree

Disagree

Neither disagree nor agree

Agree

Strongly agree

- 1. The possibility of getting infected in the future with COVID-19 concerns me.

Strongly disagree

Disagree

Neither disagree nor agree

Agree

Strongly agree

- 1. I don’t really worry about getting infected with COVID-19.

Strongly disagree

Disagree

Neither disagree nor agree

Agree

Strongly agree

- 1. I don’t think I will die if I get sick from COVID-19.

Strongly disagree

Disagree

Neither disagree nor agree

Agree

Strongly agree

- 1. I am afraid that I may die if I contract COVID-19.

Strongly disagree

Disagree

Neither disagree nor agree

Agree

Strongly agree

- 1. I am at greater risk of dying if I contract COVID-19 because of my general health.

Strongly disagree

Disagree

Neither disagree nor agree

Agree

Strongly agree

- 1. If I got infected or re-infected with COVID-19, it could be threatening to my physical health.

Strongly disagree

Disagree

Neither disagree nor agree

Agree

Strongly agree

**In order to prevent catching COVID-19, the public has been advised to do certain behaviors. For each item below, please indicate how effective you think each behavior is for protecting you from being infected with COVID-19.**

1. Wearing a mask any time you leave the house to go out in public.

Not effective at all

A little effective

Somewhat effective

Very effective

Extremely effective

1. Practicing social distancing by leaving at least six feet between you and other people (this does not include people you live with)

Not effective at all

A little effective

Somewhat effective

Very effective

Extremely effective

1. Frequently washing your hands with hand sanitizer or with warm water and soap for 20 seconds.

Not effective at all

A little effective

Somewhat effective

Very effective

Extremely effective

For each item below, please indicate how effective you think each behavior is to prevent you from spreading COVID-19 to other people.

1. Wearing a mask any time you leave the house to go out in public.

Not effective at all

A little effective

Somewhat effective

Very effective

Extremely effective

1. Practicing social distancing by leaving at least six feet between you and other people (this does not include people you live with).

Not effective at all

A little effective

Somewhat effective

Very effective

Extremely effective

1. Covering your mouth when you cough.

Not effective at all

A little effective

Somewhat effective

Very effective

Extremely effective

How often do you currently do the following?

1. Wear a mask when you leave the house to go out in public.

Always or Almost Always

Frequently

Sometimes

Rarely

Never or Almost Never

1. Practice social distancing by leaving at least six feet between you and other people (this does not include people you live with).

Always or Almost Always

Frequently

Sometimes

Rarely

Never or Almost Never

**These next questions ask about your opinions regarding COVID-19 vaccines. Please read each statement and indicate how much you agree or disagree with it by selecting the appropriate response**

1. COVID-19 vaccines are important for my health.

Strongly disagree

Disagree

Neither disagree nor agree

Agree

Strongly agree

1. Getting a COVID-19 vaccine is a good way to protect me from coronavirus disease.

Strongly disagree

Disagree

Neither disagree nor agree

Agree

Strongly agree

1. Any COVID-19 vaccine approved by the FDA and recommended by the CDC is effective.

Strongly disagree

Disagree

Neither disagree nor agree

Agree

Strongly agree

1. Getting a COVID-19 vaccine is important for the health of others in my community.

Strongly disagree

Disagree

Neither disagree nor agree

Agree

Strongly agree

1. A COVID-19 vaccine is beneﬁcial to me.

Strongly disagree

Disagree

Neither disagree nor agree

Agree

Strongly agree

1. I do what my doctor or health care provider recommends about a COVID-19 vaccine.

Strongly disagree

Disagree

Neither disagree nor agree

Agree

Strongly agree

1. COVID-19 vaccines have not been around long enough to be sure they are safe.

Strongly disagree

Disagree

Neither disagree nor agree

Agree

Strongly agree

1. I am concerned about serious side effects of COVID-19 vaccines.

Strongly disagree

Disagree

Neither disagree nor agree

Agree

Strongly agree

1. I think COVID-19 vaccines might cause lasting health problems for me.

Strongly disagree

Disagree

Neither disagree nor agree

Agree

Strongly agree

1. The information I receive about COVID-19 vaccines from my healthcare provider is reliable and trustworthy.

Strongly disagree

Disagree

Neither disagree nor agree

Agree

Strongly agree

The CDC provides trustworthy information on COVID-19 vaccines.

Strongly disagree

Disagree

Neither disagree nor agree

Agree

Strongly agree

1. I trust COVID-19 vaccines because medical organizations recommend them.

Strongly disagree

Disagree

Neither disagree nor agree

Agree

Strongly agree

**Below is a list of reasons that people consider when deciding to get the COVID-19 vaccine. Please indicate how important each of these were or could be in your decision-making.**

1. Getting a vaccine makes me personally less likely to get COVID-19.

Not at all important

Slightly important

Moderately important

Very important

Extremely important

1. Getting a vaccine makes me personally less likely to get severely sick from COVID-19.

Not at all important

Slightly important

Moderately important

Very important

Extremely important

1. Getting a vaccine makes me less likely to give COVID-19 to my family.

Not at all important

Slightly important

Moderately important

Very important

Extremely important

1. It is good for the health of the community for me to get the vaccine.

Not at all important

Slightly important

Moderately important

Very important

Extremely important

1. Getting a vaccine allows me to return to my normal activities.

Not at all important

Slightly important

Moderately important

Very important

Extremely important

1. Getting a vaccine helps the economy get back to normal.

Not at all important

Slightly important

Moderately important

Very important

Extremely important

1. Getting a vaccine allows me to wear a mask less.

Not at all important

Slightly important

Moderately important

Very important

Extremely important

1. Getting a vaccine allows me to social distance less.

Not at all important

Slightly important

Moderately important

Very important

Extremely important
